# Genetic variation in glutamatergic genes moderates the effects of childhood adversity on brain volume and IQ in treatment-resistant schizophrenia

**DOI:** 10.1038/s41537-023-00381-w

**Published:** 2023-09-14

**Authors:** Suriati Mohamed Saini, Chad A. Bousman, Serafino G. Mancuso, Vanessa Cropley, Tamsyn E. Van Rheenen, Rhoshel K. Lenroot, Jason Bruggemann, Cynthia S. Weickert, Thomas W. Weickert, Suresh Sundram, Ian P. Everall, Christos Pantelis

**Affiliations:** 1 Department of Psychiatry, Faculty of Medicine, Universiti Kebangsaan Malaysia, Jalan Yaacob Latif, Cheras, Kuala Lumpur Malaysia; 2 Department of Psychiatry, Hospital Canselor Tuanku Muhriz, Jalan Yaacob Latif, Cheras, Kuala Lumpur Malaysia; 3 Melbourne Neuropsychiatry Centre, Department of Psychiatry, The University of Melbourne and Melbourne Health, Parkville, VIC Australia; 4 Alberta Children’s Hospital Research Institute, Calgary, AB Canada; 5 Hotchkiss Brain Institute, University of Calgary, Calgary, AB Canada; 6 Department of Medical Genetics, Psychiatry, and Physiology and Pharmacology, The University of Calgary, Calgary, AB Canada; 7 Centre for Mental Health, Faculty of Health, Arts and Design, Swinburne University, Melbourne, VIC Australia; 8 School of Psychiatry, University of New South Wales, Randwick, NSW Australia; 9 Department of Psychiatry and Behavioural Science, University of New Mexico, Albuquerque, NM USA; 10 Neuroscience Research Australia, Randwick, NSW Australia; 11 Schizophrenia Research Institute, Sydney, NSW Australia; 12 Department of Neuroscience & Physiology, SUNY Upstate Medical University, NY, USA; 13 Schizophrenia Research Laboratory, Neuroscience Research Australia, NSW, Australia; 14 Department of Psychiatry, School of Clinical Sciences, Monash University, Clayton, VIC Australia; 15 Monash Medical Centre, Monash Health, Clayton, VIC Australia; 16 Florey Institute of Neuroscience and Mental Health, The University of Melbourne, Parkville, VIC Australia; 17 Institute of Psychiatry, Psychology & Neuroscience, King’s College London, London, UK; 18 Western Centre for Health Research & Education, Sunshine Hospital, Western Health, St Albans, VIC 3021 Australia

**Keywords:** Developmental biology, Schizophrenia

## Introduction

Childhood adversity describes a range of adverse experiences, including sexual, physical, and emotional abuse, neglect, and other adverse life events that have occurred during childhood^1^. It is associated with an elevated risk of schizophrenia^1,2^. Studies of individuals with childhood adversity and schizophrenia report lower intelligence quotient (IQ) scores^3,4^. A population twins study found that children exposed to domestic violence had lower IQs than unexposed children, regardless of underlying genetic factors^3^. Meanwhile, another study on 216 twins including monozygotic (MZ) and dizygotic (DZ) probands pairs and MZ/DZ healthy controls showed that schizophrenia polygenic risk score (PRS) and childhood trauma predict schizophrenia vulnerability^2^. Given that schizophrenia has also continuously been linked to lower IQ levels as two meta-analysis studies reported that the mean IQ scores of individuals who subsequently develop schizophrenia are lower than those of healthy comparison individuals years before the beginning of psychotic symptoms^5,6^, the impact of childhood abuse on cognition may constitute a step in the developmental process leading to psychosis^7^. Low IQ in patients with schizophrenia has been identified as a predictor of poor social and clinical outcomes^8^. Fundamental questions that are highly relevant to our understanding of schizophrenia, and as yet unresolved, are whether the effect of childhood adversity on IQ is influenced by brain volume or genetic variation and if so, does the effect of these factors differ between patients with and without treatment-resistant schizophrenia (TRS)?

In the present study, we sought to examine potential mediating (i.e., total brain volume [TBV]) and moderating (i.e., glutamatergic genetic risk score [GRS]) factors underlying this complex relationship. A mediator is a third variable that explains, fully or in part, the relationship between an independent variable and a dependent variable^9^. Whereas, a moderator is a variable that affects the direction and/or strength of the relationship between an independent variable (i.e., childhood adversity) and a dependent variable (i.e., IQ)^9^.

### The moderating role of glutamatergic genes

Glutamate is involved in cell proliferation, migration, differentiation or survival, and synapse formation during brain development^10^. Cortical expression of most glutamate subunit receptors in the human neocortex and cerebellum begins during the embryonic period, peaks during early foetal life, and remains highly expressed postnatally^11^. The Schizophrenia Working Group of the Psychiatric Genomics Consortium (PGC) genome-wide association study (GWAS) 2 highlighted associations between genetic variation in glutamate genes and schizophrenia, specifically, single-nucleotide polymorphisms (SNPs) in *GRM3* (metabotropic glutamate receptor 3)*, GRIN2A* (*N*-methyl-d-aspartate [NMDA] type subunit GluN2A)*, SRR* (serine racemase)*, GRIA1* (GluA1 subunit of α-amino-3-hydroxy-5-methyl-4-isoxazole propionic acid [AMPA] receptor), and *GPM6A* (glycoprotein M6A), among which *GRM3* rs1270429*0* is one of the top hits^12^. The latest PGC GWAS fine-mapping showed that the glutamate receptor genes are strongly implicated in individuals with schizophrenia specifically *GRIN2A*, *GPM6A* and *GRM1* (metabotropic glutamate receptor 1)^13^. In addition, a recent meta-analysis using the whole exomes of 24,248 schizophrenia cases and 97,322 controls found rare coding variants in 10 genes confer substantial risk for individuals with schizophrenia which include genes in the glutamatergic system^14^.

Glutamatergic genes are associated with brain morphology^15^ and cognition^16^ in schizophrenia. Disruption of glutamate signalling has been linked to working memory deficits in individuals with schizophrenia^17^. A recent study found that glutamatergic polygenic risk score (PRS) is linked with attention and brain activity during variable attention control in schizophrenia^18^. In addition, PRS for schizophrenia is associated with lower general cognitive ability in individuals with schizophrenia compared to controls^19^.

Moreover, chronic stress is associated with the loss of glutamate receptors and suppression of glutamate transmission^20^, supporting the notion that abnormalities in glutamatergic neurotransmission in the brain play a crucial role in the adverse effects of stress in schizophrenia^21^. Thus, it is possible that glutamate may influence brain volume and cognition in schizophrenia through its interaction with childhood adversity, which is highly prevalent among those with the illness.

### Why is it important to study TRS?

TRS affects approximately 30% of schizophrenia patients^22,23^. TRS is defined as a non-response to at least two sufficient trials of antipsychotic therapy. Evidence suggests that individuals with TRS tend to have multiple risk factors such as neurodevelopmental factors, childhood adversity, genetics, substance use, comorbidities, etc.^24,25^. A study by Hassan and De^26^ reported that TRS patients had higher scores on both childhood trauma and adverse life events.

In addition, a systematic review reported that relative to individuals who responded to treatment, TRS patients are categorically distinct from treatment-responsive schizophrenia by exhibiting glutamatergic abnormalities, a lack of dopaminergic abnormalities, and significant reductions in grey matter and had a higher family history loading^27^. Higher level of glutamate in the anterior cingulate is found in TRS compared to non-TRS patients^28^. TRS patients demonstrated significantly higher total glutamate + glutamine (Glx) levels in the putamen than both treatment-responsive and ultra-TRS patients^29^. Anderson et al.^30^ found that TRS and ultra-TRS patients demonstrated considerably reduced grey matter volumes compared to non-TRS patients and healthy controls.

### The present study

**Fig. 1:**
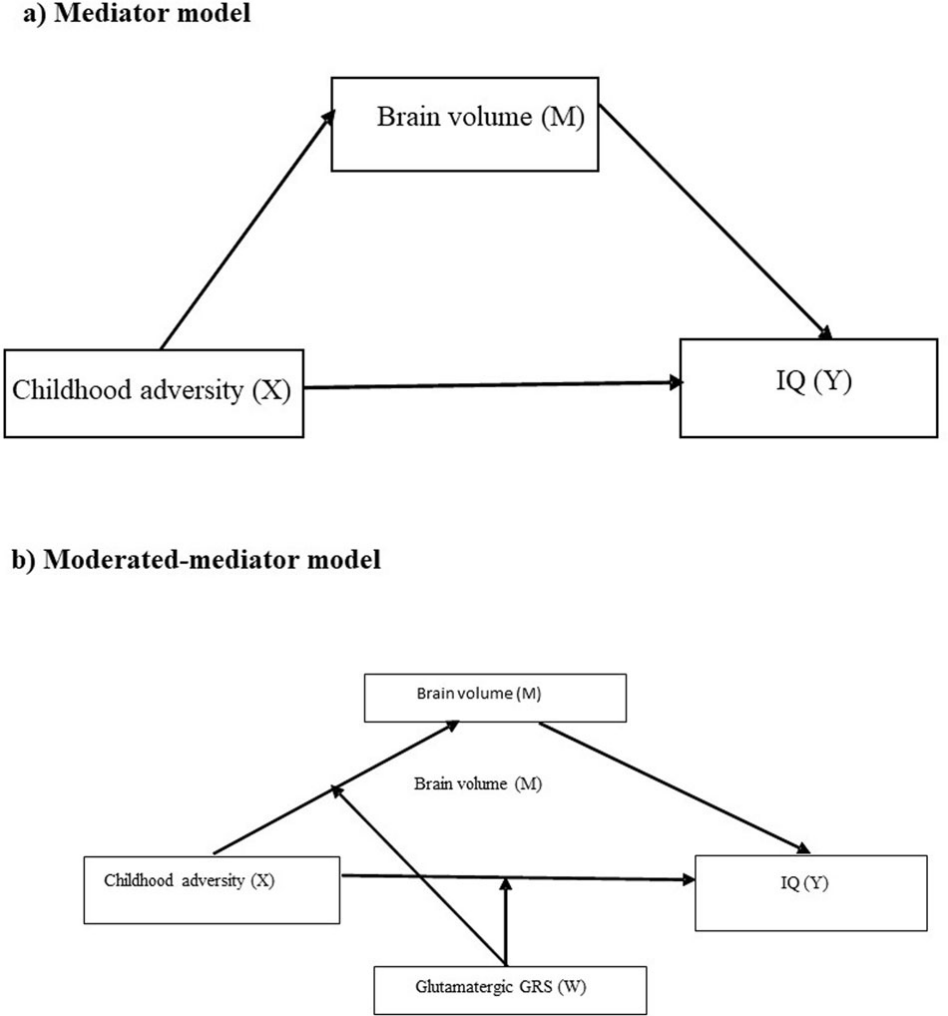
Hypothetical models for the study. **a** Mediator model and **b** Moderated-mediator model.

In this study, we aimed to examine the extent to which the association between childhood adversity and IQ was moderated by genetic variation in genes involved in glutamate signalling, in the context of TBV as a mediator (moderated-mediation model) using cohorts of patients with TRS and treatment-responsive schizophrenia (Fig. 1a, b). We hypothesised that glutamatergic GRS would moderate the effect of childhood adversity on TBV and IQ in the TRS cohort due to previous work suggesting TRS patients have more marked glutamatergic abnormalities^27,31^.

## Methods and materials

### Participants

TRS cohort (CRC-TRS): Data for the TRS cohort was obtained from Cooperative Research Centres for Mental Health – Psychosis Study (CRC). The participants in this CRC study included inpatient and outpatient clinics located in Melbourne, Australia. The inclusion criteria for TRS participants included: (i) a diagnosis of schizophrenia or schizoaffective disorder, (ii) currently or previously prescribed and/or recommended for clozapine treatment, and (iii) aged between 18 and 65 years. The Mini Neuropsychiatric Interview (MINI)^32^ was administered to all participants to confirm the diagnosis, as well as to rule out current or past psychiatric illness in healthy controls. Control subjects with a personal or family history of psychiatric disorder, neurological disorder, head injury, prior or current use of antipsychotics, thyroid dysfunction and substance use disorder were excluded. All participants gave informed consent, and the study was approved by the Melbourne Health Human Research Ethics Committee (MHREC ID 2012.069).

Schizophrenia cohort (ASRB-Scz): Genotype, childhood adversity, IQ and TBV data were obtained from the Australian Schizophrenia Research Bank (ASRB)^33^ for the schizophrenia cohort. The ASRB is an established, registered multisite collaborative project across five Australian states and territories^33^. Participants were recruited if they were fluent in English and aged between 16 and 65 years. Diagnosis of schizophrenia or schizoaffective disorder was confirmed using the OPCRIT algorithm applied to interviewer ratings on the Diagnostic Interview of Psychosis (DIP), which is based on the Diagnostic and Statistical Manual of Mental Disorders, Fourth Edition (DSM IV) and the 10th revision of the International Statistical Classification of Diseases and Related Health Problems (ICD-10) criteria. For this study, patients on clozapine medication were excluded.

### Childhood adversity

In the CRC-TRS cohort, childhood adversity was measured using the Childhood Trauma Questionnaire-Short Form (CTQ-SF)^34^. The CTQ-SF is a 28-item self-report questionnaire that retrospectively measures five types of maltreatment, including physical, sexual, and emotional abuse, and emotional and physical neglect, before 18 years of age. Items were rated on a 5-point Likert scale (1 = “never true” to 5 = “very often true”) and summed into a total score, where higher scores are indicative of more severe childhood adversity exposure.

In the ASRB-Scz cohort, childhood adversity was measured using the Childhood Adversity Questionnaire (CAQ). The CAQ is a 21-item self-report questionnaire that retrospectively asks about childhood experiences before 18 years of age, including perceptions of lack of affection, nervous or emotional trouble, drinking or other drug use in parent figures, household conflict, parental separation or divorce, childhood happiness, parental indifference, neglect, authoritarian or normal upbringing, financial hardship, parental emotional, physical, and sexual abuse and other types of mistreatment^35^. Higher scores indicate greater adversity.

A systematic review study shows that the CTQ exhibits strong evidence for internal consistency, reliability, content validity, structural validity, and convergent validity^36^. Meanwhile, the CAQ demonstrates only moderate evidence for internal consistency^36^.

### Intelligence quotient

IQ was assessed using the Wechsler Abbreviated Scale of Intelligence (WASI)^37^ for both ASRB-Scz and CRC-TRS cohorts. The WASI consists of four subtests (Vocabulary, Block Design, Similarities, and Matrix Reasoning). To provide an estimate of current intelligence, the age-standardised scores based on the performance on these four subscales were calculated.

### Genotyping and risk score calculation

For the CRC-TRS cohort, DNA was extracted from blood samples and genotyped using the Agena MassARRAY MALDI-TOF genotyping system (Agena, Inc., San Diego, CA). Meanwhile, in the ASRB-Scz cohort, DNA was obtained from whole blood using a QIAamp DNA Blood Midi or Maxi Kit (QIAGEN, Chadstone, Australia) as directed by the manufacturer’s instructions, and genotyped using the Illumina Goldengate assay (San Diego, CA). In total, 22 SNPs within six glutamatergic genes (*GRM3, GRIN2A, GRIA1, GRIK3, GPM6A* and *SSR*) were previously shown to be associated (*p* < 0.05) with schizophrenia by the Schizophrenia Working Group of the PGC, were selected. Following linkage disequilibrium (LD) analysis, one SNP with the lowest *p* value in the PGC GWAS study^12^ was selected from each LD block (*D* > 0.8) (Supplementary Fig. S1). In total, 15 SNPs were carried forward to analysis (*GRM3*: rs13242038, rs757656, rs12704290, rs2228595, rs1989796; *SRR*: rs4523957; *GRIN2A*: rs9922678; *GRIA1*: rs707176; *GRIK3*: rs535620, rs6671364, rs6691840, rs17461259, rs472188; and GPM6A: rs10520303, rs1106568). Prior to analysis, PLINK v1.90b4 was used to calculate a weighted glutamatergic risk score (GRS), where the schizophrenia risk alleles carried by an individual were weighted by their corresponding log-odds ratio from the PGC GWAS study^12^ and then summed.

### Neuroimaging

In the CRC-TRS cohort, a high-resolution T_1_-weighted structural MRI (Magnetisation-Prepared Rapid Acquisition Gradient Echo) sequence was incorporated on Siemens Avanto 3 T Magnetom TIM Trio scanner using a standard data acquisition (176 contiguous 1 mm sagittal slices; field-of-view 250 × 250 mm^2^, repetition time/echo time = 1980/4.3 ms, data acquisition matrix 256 × 256, voxel dimensions = 0.98 × 0.98 × 1.0 mm^3^, flip angle 15°).

For the ASRB-Scz cohort, a high-resolution T_1_-weighted structural MRI scan (MPRAGE) sequence was incorporated on Siemens Avanto 1.5 Tesla scanners using a standard data acquisition protocol (176 contiguous 1 mm sagittal slices; field-of-view 250 × 250 mm^2^, repetition time/echo time = 1980/4.3 ms, data acquisition matrix 256 × 256, voxel dimensions = 0.98 × 0.98 × 1.0 mm^3^, flip angle 15°) across all ASRB sites^33^.

### Freesurfer processing and quality control

Image processing was conducted using the Freesurfer software package (version 5.1.0, http://surfer.nmr.mgh.harvard.edu/)^38–41^. The automated volume-based stream was used to obtain mean volume estimates across the entire brain for TBV (brain segmentation volume without ventricles). Freesurfer’s editing tools were used to correct errors in outlining brain structures in compliance with internal, standardised quality control and editing protocol.

For quality control, each (raw) scan was inspected for errors in a viewer such as MIPAV or Osirix. A four-point scale was used, where 1 and 2 were considered usable (artefact was perceptible but very small); 3 had some usable regions but would not be included in routine data sets; 4 was unusable. Edited images were then reprocessed, and the output visually re-inspected. This process was repeated until all surface errors were fixed, and any images that failed this process were omitted from the analysis. The ASRB data came with a spreadsheet that indicated whether specific scans had an artefact or not which included motion, contrast, orientation, radiofrequency (RF), and gross brain abnormalities. Quality control was based on the Alzheimer’s Disease Neuroimaging Initiative (ADNI) protocol. Scans were given a score of Global Pass, Global Fail, or Partial Fail, with a list of regions that were not corrected.

The Freesurfer processing and manual correction were executed by four trained raters, blind to diagnosis. Inter-rater reliability of the final volume estimates (after correction) was calculated for 34 brain regions from a subset of 20 volumes. The intra-class coefficient (ICC) was >0.90 for all regions except for the left (0.72) and right (0.59) temporal pole and the left (0.81) and right (0.82) frontal pole. In total, 114 scans were excluded due to artefacts and other factors^42^.

### Statistical analysis

SPSS version 28 software for Window® (SPSS Inc., Chicago, USA) was utilised for statistical analyses. Demographic and clinical characteristics were analysed using independent t-tests (continuous variables) or chi-square tests (categorical variables). Before model testing, multivariate outliers were evaluated using Mahalanobis distance with a *p* < 0.001 criteria. Assumptions concerning homoscedasticity, linearity of the relationships between dependent and independent variables, and normality of residuals were evaluated using SPSS software and were fulfilled in all models.

We performed moderated-mediation analysis using model 10 in the SPSS PROCESS macro for SPSS version 3.0^43^, where diagnosis (*W*) was used as the moderator in the pooled sample of patients and controls to test the diagnosis effect, with 5000 bias-corrected bootstrap samples. By using bias-corrected bootstrap confidence intervals (CIs) with a single test procedure to specifically test the indirect effect of an independent variable (*X*) on a dependent variable (*Y*) through a mediator (*M*), the PROCESS macro provides a more powerful approach than Baron and Kenny^22^ traditional causal steps^43^. Mediation was considered to be present if paths *X* to *M* and *M* to *Y* were significant, and *c*’ (effect of *X* on *Y* when *M* was entered simultaneously) was smaller than *X* to *Y* (denoted as *c*) by a non-trivial amount^43^. Mediation was significant when the 95% CIs of the indirect effects did not include zero.

In the case of significant interactions between diagnosis and other key variables, mediation and moderated-mediation analyses stratified by diagnosis were performed. A moderated-mediation model (PROCESS model 8) was utilised to examine the association between childhood adversity (*X*) and IQ (*Y*) in the context of TBV as a mediator (*M*) and GRS as a moderator (*W*) in stratified samples of schizophrenia patients and healthy controls. We chose this multilevel model for mediation and moderated-mediation analyses because it shifts point estimates and their respective intervals towards each other, meaning that the intervals for comparisons are more likely to include zero, and are therefore more likely to be valid^44^. Post hoc graphs of the significant moderation analyses were constructed a median split of the glutamatergic GRS. GRS values less than the median were labelled “Low GRS”, whereas values more than the median were labelled “High GRS”. The moderated-mediation analysis was utilised as compared to other alternative analyses reflect the maturation of scientific research to address the specifics of how the exposures have their effects.

## Results

### Participant characteristics

Participant characteristics for CRC-TRS and ASRB-Scz cohorts are presented in Table 1. Schizophrenia and TRS patients had significantly higher childhood adversity (*p* < 0.001) and lower IQ performance compared to healthy controls (*p* < 0.001 in the ASRB-Scz and *p* < 0.001 in the CRC-TRS cohorts). CRC-TRS patients had significantly lower mean IQ than the ASRB-Scz patients. In addition, the CRC-TRS patients had higher mean glutamatergic GRS and lower mean TBV than the ASRB-Scz patients and healthy controls. However, the differences between the mean scores of glutamatergic GRS and TBV in the CRC-TRS and ASRB-Scz were not statistically significant.

**Table 1:** Demographic and clinical characteristics of the study cohorts.

Variables	CRC-TRS		ASRB-Scz		Comparisons, *p* value			
	(A) Controls *n* = 40	(B) TRS *n* = 51	(C) Controls *n* = 118	(D) Schizophrenia *n* = 154	A vs B	A vs C	B vs D	C vs D
Age, mean, years (SD)	39.1 (10.8)	39.5 (1.3)	41.1 (13.7)	37.3 (9.91)	0.912	0.181	0.075	0.010
Gender, male, *n* (%)	24 (39.3)	37 (60.7)	58 (49.2)	107 (69.5)	0.117	0.235	0.587	<0.001
Childhood adversity, mean (SD)	30.7 (4.9)	42.3 (14.2)	2.8 (3.3)	5.8 (4.7)	<0.001	0.01	<0.001	<0.001
Childhood adversity using *z*-score, mean (SD)	−0.5 (0.3)	0.4 (1.2)	−0.4 (0.8)	0.3 (1.1)	<0.001	<0.001	0.627	<0.001
GRS, mean (SD)	0.0005 (0.0028)	0.0012 (0.0029)	0.0006 (0.0031)	0.0009 (0.0028)	0.508	0.620	0.432	0.365
Duration of illness, mean (SD)		No data available		13.9 (8.9)				
WASI, mean IQ (SD)	111.7 (10.7)	86.1 (18.3)	116.7 (11.4)	106.27(13.5)	<0.001	0.410	0.005	<0.001
TBV, mean (SD)^a^	1140.5 (1262.8)	1100.41 (121.1)	1162.7 (117.9)	1183.7 (116.0)	0.741	0.601	0.350	0.072

*GRS* glutamatergic genetic risk score, *SD* standard deviation, *TRS* treatment-resistant schizophrenia, *WASI* Wechsler Abbreviated Scale of Intelligence.

^a^Brain volume (uncorrected) (mm^3^).

### Moderated-mediation findings

**Fig. 2:**
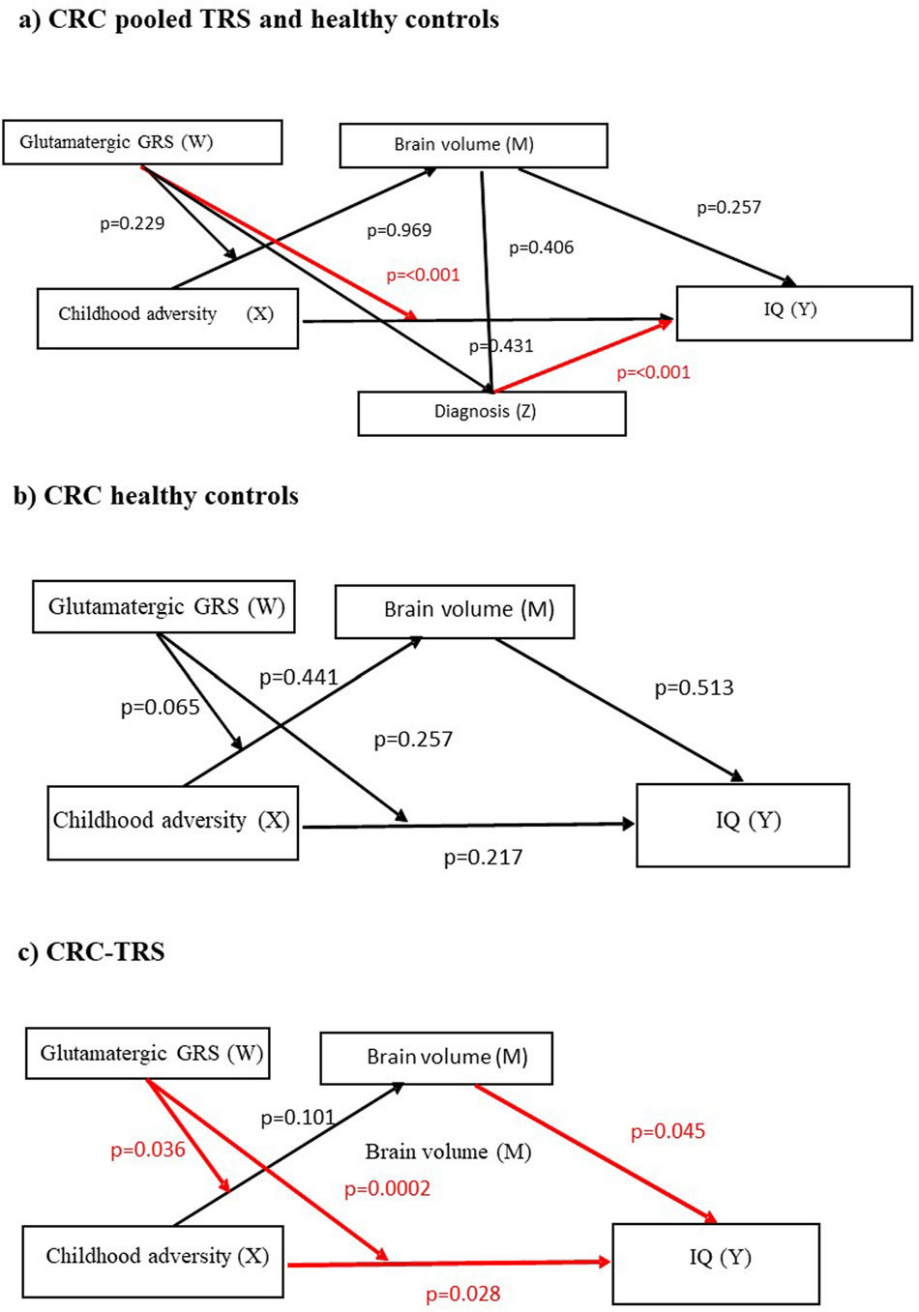
Moderated-mediation analysis of the relationship between childhood adversity glutamatergic GRS, TBV and IQ. **a** CRC pooled TRS and healthy controls detected a significant moderation effect of glutamatergic GRS on the association between childhood adversity and IQ (*p* < 0.001), as well as a significant direct association between diagnosis and IQ (*p* < 0.001), **b** CRC-TRS healthy controls group did not show any significant association **c** CRC-TRS group revealed that glutamatergic GRS moderated the association between childhood adversity and TBV (*p* = 0.036), as well as the association between childhood adversity and IQ (*p* = 0.0002). There was also a direct association between childhood adversity and IQ (*p* = 0.028), and TBV with IQ (*p* = 0.045).

Analysis of a pooled sample of cases and controls in the CRC-TRS cohort detected a significant moderation effect of glutamatergic GRS on the association between childhood adversity and IQ (*β* = −138.64, *p* < 0.001, 95% CI = −193.73 to −83.55; Fig. 2a). There was also a significant direct association between diagnosis and IQ (*β* = −26.27, *p* < 0.001, 95% CI = 35.09 to −17.45; Fig. 2a).

**Fig. 3:**
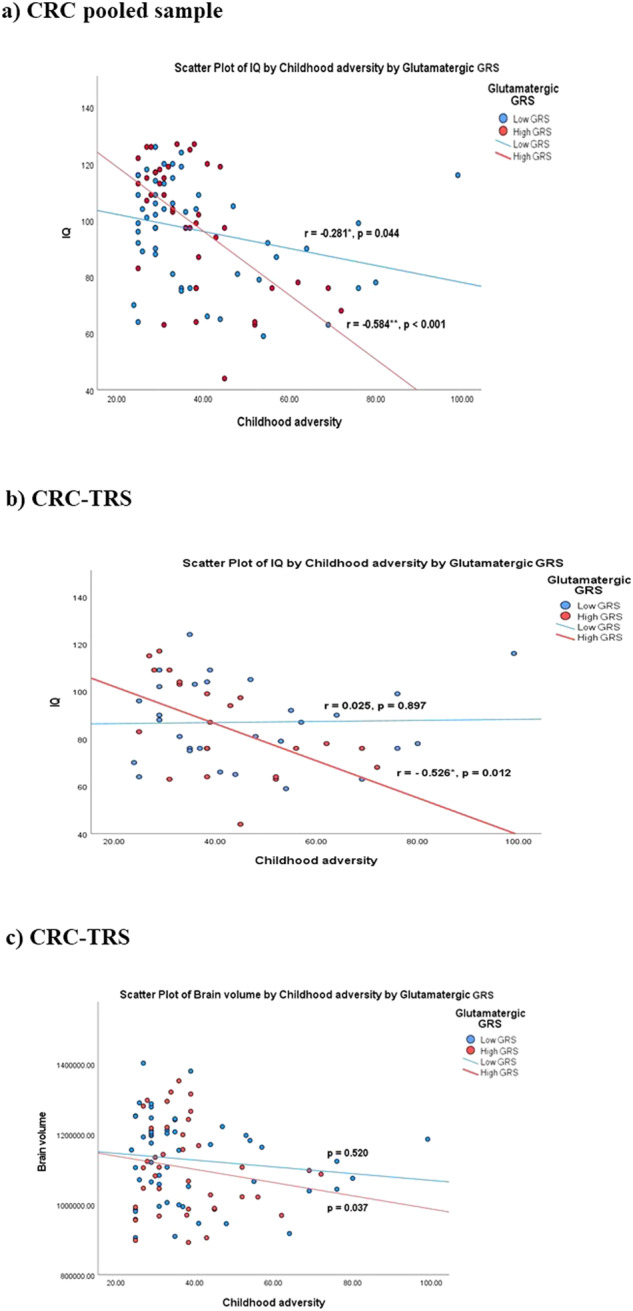
Scatter plot graphs of the moderation effect of glutamatergic GRS on the association between childhood adversity and IQ and TBV. **a** Pooled sample of healthy controls and TRS showed that those with high childhood adversity score and high GRS had lower IQ performance (*r* = –0.584, *p* < 0.001), **b** participants with high childhood adversity score and high GRS had lower IQ performance (*r* = –0.526, *p* < 0.012) in TRS group and **c** participants with high childhood adversity score and high GRS had lower TBV (*p* < 0.037) in TRS group.

Stratified analyses by diagnosis revealed that glutamatergic GRS moderated the association between childhood adversity and TBV (*β* = −543.27, *p* = 0.036, 95% CI = −1048.56 to −37.99) as well as the association between childhood adversity and IQ (*β* = −125.05, *p* = 0.0002, 95% CI = −188.16 to −61.94) in the TRS patients (Fig. 2c) but not healthy controls (Fig. 2b). In addition, there were significant direct associations between childhood adversity and IQ (*β* = −0.32, *p* = 0.028, 95% CI = −0.601 to −0.04) as well as TBV and IQ (*β* = 0.04, *p* = 0.045, 95% CI = 0.001 to 0.078) in the cases but not controls. Post hoc analysis showed childhood adversity was negatively correlated with IQ among cases with a high GRS (Fig. 3a, b). Likewise, a negative association between childhood adversity and TBV was only detected among cases with a high GRS (Fig. 3c).

**Fig. 4:**
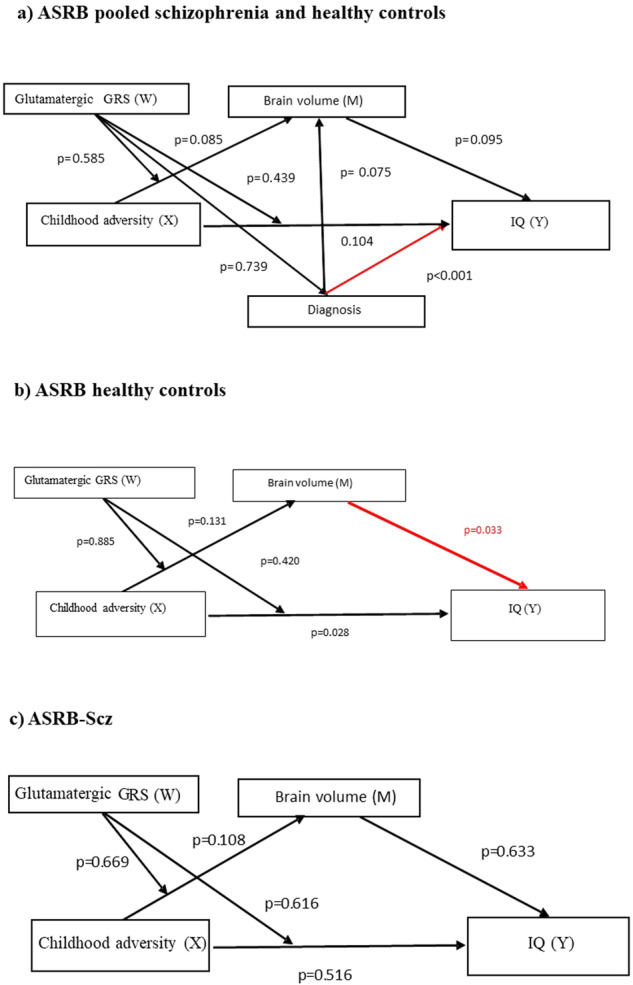
Moderated-mediation analysis of the relationship between childhood adversity glutamatergic GRS, TBV and IQ. **a** Pooled cases and controls in the ASRB-Scz cohort revealed a direct association between diagnosis and IQ (*p* < 0.001), **b** ASRB healthy controls group showed a direct association between TBV and IQ (*p* = 0.033), and **c** ASRB-Scz group did not show any association.

Examination of pooled cases and controls in the ASRB-Scz cohort revealed a direct association between diagnosis and IQ (*β* = −9.16, *p* < 0.001, 95% CI = −12.77 to −5.55) (Fig. 4a). Stratified analysis by diagnosis showed a direct association between TBV and IQ (*β* = 0.02, *p* = 0.033, 95% CI = 0.002 to 0.036) in healthy controls (Fig. 4b). However, no direct association was found between TBV and IQ among ASRB-Scz patients (Fig. 4c).

## Discussion

The finding of this study supports our hypothesis that the association between childhood adversity and TBV as well as IQ in TRS is moderated by genetic variation in genes involved in glutamate signalling. TRS patients with higher childhood adversity and higher glutamatergic GRS performed lower in IQ and had lower TBV. However, these effects were not observed in non-TRS schizophrenia patients (ASRB-Scz) and healthy control groups. These findings indicate that the alteration of brain structure and cognition in TRS is derived from the interacting influence of childhood adversity and variation in genes associated with glutamate signalling. These effects likely result from a pathological process of brain development during pre and post-puberty^45^.

The aetiology and pathophysiology of schizophrenia are believed to involve both early (during the pre-and perinatal periods) and late (during the adolescence period) neurodevelopmental processes^46^. In the case of early neurodevelopmental processes, brain abnormalities appear to be in part, the result of an inherited vulnerability state that manifests prior to illness onset^47,48^. The cortical development involves the formation and organisation of glutamatergic (excitatory neurotransmitter) as well as gamma-aminobutyric acid (inhibitory) neurons. Early disturbance of these intricate processes and cellular signals, as seen in schizophrenia, is hypothesised to cause the improper formation of the precise connections required for healthy, functional neural networks^47^. Glutamatergic dysfunction has been implicated in the pathophysiology of TRS. Two studies found higher glutamate levels in the pregenual anterior cingulate cortex (pgACC) in TRS patients, one in comparison to healthy controls^49^ and the other in comparison to patients who responded to non-clozapine antipsychotics^28^. Another study found that clozapine-resistant individuals with TRS had higher glutamine (Glx) levels in the dorsal ACC compared to healthy controls^50^. At the electron microscope (EM) level, TRS patients were found to have more excitatory synapses in limbic striatal regions in comparison to treatment responders^51^. Although genetic factors constitute a significant contributor to the disease, the presence of genetic predisposing alone may not be sufficient for the manifestation of schizophrenia^46,52^. The transmission pattern of schizophrenia is complex, involving genes as well as environmental influences^52^. The pathological processes underlying such progressive neural changes during “late neurodevelopment” may reflect anomalies in synaptic plasticity^46^. The cortical thinning and global cognitive decline during the course of schizophrenia could be explained by a pathologically prolonged process of synaptic elimination or pruning^53,54^.

Synaptic pruning is the process of elimination of extra neural synapses. A review by Pantelis et al.^46^ on the longitudinal and cross-sectional imaging studies, as well as animal studies, proposed that schizophrenia may arise from abnormalities or insults at various stages of brain maturation, involving the interaction of genetic influences and early (during foetal development) and later insults (during childhood or adolescence). The impact of such interactions and the timing of illness onset may influence the features observed, impact developmental trajectories and provide some explanation for the heterogeneity observed in schizophrenia^55,56^. Thus, there is evidence of accelerated grey matter loss across the cortex representing a twofold accelerated thinning of grey matter, which is most apparent in the prefrontal lobe with illness onset in adolescence^51^, while more posterior regions are impacted with illness onset at the time of puberty in childhood-onset schizophrenia^57^. These abnormal neurodevelopmental processes in genetically vulnerable individuals may interact with other risk factors for psychosis such as childhood adversity (e.g. trauma) or other environmental insults (e.g. cannabis) around the time transition to active illness beginning in adolescence and early adulthood^2^, which together would have neuroprogressive consequences^46^. Chronic stress through childhood adversity can damage glial cells^58^, which contributes to reducing glial uptake of glutamate and causing the elevation of extracellular glutamate levels^59^. With its neurotoxic effects, elevated extracellular glutamate levels can further accelerate neuronal and glial damage^60^. Repeated and chronic stress exposure has been shown to significantly impair cognitive performance by suppressing α-amino-3-hydroxy-5-methyl-4-isoxazolepropionic and *N*-methyl-d-aspartate receptors synaptic transmission and glutamate receptor expression in the frontal cortex^61^. Different parts of the glutamatergic pathway also appear to be involved in different cognitive domains, such as presynaptic component synthesis and glutamate uptake involved in memory and postsynaptic signalling involved in working memory^62^. Our finding that higher glutamatergic GRS was associated with lower IQ performance and lower TBV among CRC-TRS patients in the face of higher childhood adversity suggests that glutamatergic GRS may cause detrimental effects when synergised with childhood adversity. This effect is likely developmentally determined and would suggest that TRS may be apparent early in the course of illness, though this remains to be proven. It is also plausible that the effects of glutamatergic GRS could be specific to the illness stage or greater illness severity, as seen in TRS.

We also found that CRC-TRS patients and ASRB_Scz patients had a significantly lower mean IQ than healthy controls, which is consistent with the notion that early neurodevelopmental insults would impact global cognitive ability (IQ). However, the IQ impairment is more marked in the CRC-TRS patients. This notion is further supported by our finding of a direct association between childhood adversity with IQ, and TBV with IQ in the TRS patients. Legge et al.^63^ found that lower premorbid IQ was associated with an increased risk of TRS. This study did not find evidence of an association between TRS and childhood adversity^63^. However, a previous study reported a cumulative effect of lifetime adversity in TRS^26^, which suggests that childhood adversity could be contributing to TRS in a cumulative manner^63^. A direct relationship between childhood adversity and IQ, as well as moderating effect of glutamatergic GRS on this relationship, suggests that childhood adversity may have a distinct effect on IQ in TRS than non-TRS patients.

Our study is also consistent with others that have found TRS patients to have greater cognitive deficits than non-TRS patients^64–66^ and healthy controls^67^. We also found that there was a positive association between TBV with IQ in CRC-TRS patients. Studies on antipsychotic-naïve patients with schizophrenia found a positive correlation between IQ with brain volume^68,69^, and total brain volume but not grey matter^70^. A longitudinal study by Kubota et al.^71^ that examines the association between brain measures and IQ across time in schizophrenia reported a positive correlation between IQ with cortical volume and thickness in widespread brain regions including the frontal, temporal and parietal cortices, implying that the progressive loss of brain tissue in schizophrenia is linked with IQ decline^71^. Study that specifically looks at the relationship between TBV and IQ in TRS patients is limited. A recent study by Syeda et al.^67^ discovered a TRS signature that comprised a differential cortico-cognitive pattern that included frontal and temporal lobes as well as paired associates learning and intra-extra dimensional set-shifting.

There are aspects of the current study design that potentially limit the interpretation of these findings. First, other genetic or environmental factors were not analysed in this study, and these may have influenced the effects of childhood adversity on TBV and IQ. Second, although the reliability of retrospective self-report measures in patients with psychosis is questionable, previous studies have demonstrated that retrospective self-reports of childhood adversity by schizophrenia patients are as reliable as those made by the general population, independent of the current severity of psychotic symptoms^36,72,73^. Third, we acknowledged that our risk score does not fully capture genetic variation in the glutamate signalling pathway, and therefore future functional work will be needed to validate our findings. Fourth, to establish that the glutamatergic GRS has particular significance for IQ and TBV requires a comparison of this set with random selections of SNPs not involved in glutamate function. Unfortunately, we do not have access to SNPs other than the ones presented in this manuscript. Thus, we emphasised that our approach is preliminary and will require validation before drawing firm conclusions. Finally, the minimum sample size required for 80% power in the multivariate analysis for 7 predictors as in PROCESS model 10 is 103. We admitted that the CRC-TRS cohort is slightly underpowered for 91 participants. However, this study has more genetic, imaging, cognition and childhood adversity data than other existing literature in TRS study. We believed that our findings will add value to the body of knowledge in understanding the complex relationship between childhood adversity, glutamatergic genes, TBV and IQ. Nevertheless, in overcoming this limitation, we employed the PROCESS macro with 5000 bootstrapping procedure analysis, yielding results that were more accurate and less influenced by sample size^74–76^.

In conclusion, our study found a synergistic effect between childhood adversity and glutamatergic GRS on TBV and IQ in CRC-TRS patients, but not in ASRB-Scz and healthy controls. Future efforts to extend our results and expand our understanding of the interplay between genetic and environmental factors are warranted and could, if replicated, inform early detection and interventions to prevent the downstream consequences of childhood adversity.

### Supplementary information

Supplementary materials

## Data Availability

The author states that the data will be available upon request.
