# Supplementary material for: Genetic variation in glutamatergic genes moderates the effects of childhood adversity on brain volume and IQ in treatment-resistant schizophrenia
Source: Schizophrenia (Heidelb). 2023 Sep 14;9(1):59. doi: 10.1038/s41537-023-00381-w (PMC10502098; doi:10.1038/s41537-023-00381-w)
Supplement: Supplementary file 1 — Supplementary materials [file 41537_2023_381_MOESM1_ESM.docx]

**Table of contents**

[Figure S1: Schematic picture of haplotype structure. 2](#_Toc106956990)

[Figure S2: Histogram of the mean glutamatergic GRS by diagnosis in both ASRB and CRC cohorts. 3](#_Toc106956991)

1. ***GRM3***  b) ***GRIK 3***


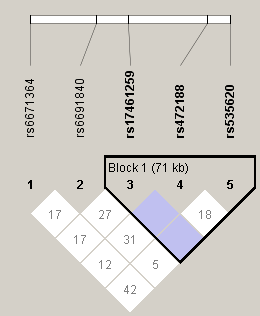

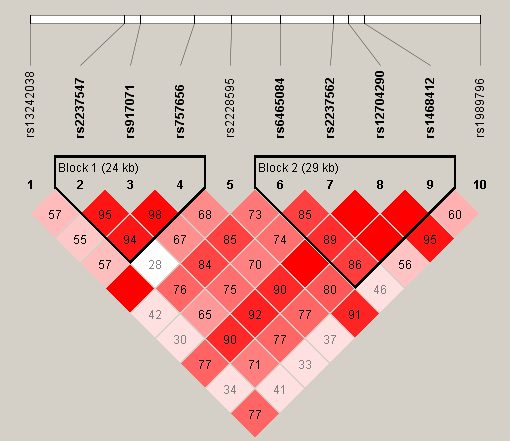


c) ***GPM6A***  d) ***SRR***  e) ***GRIA1***


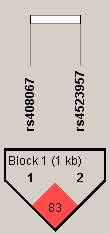

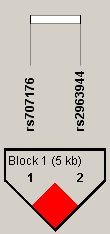

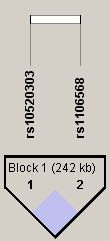


Figure S1: Schematic picture of haplotype structure.

The haploblock structures were generated using a solid spine of linkage disequilibrium (LD), with the defaulted setting of D’ > 0.8.


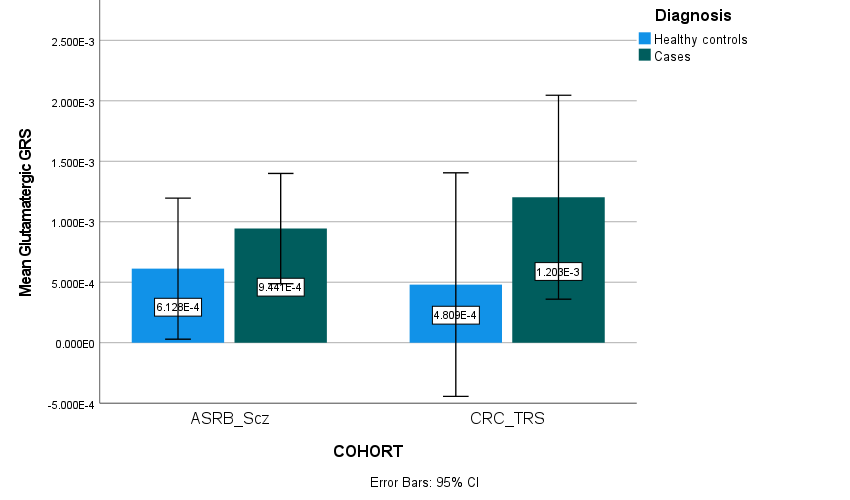


Figure S2: Histogram of the mean glutamatergic GRS by diagnosis in both ASRB and CRC cohorts.
